# Epigenome-Wide Associations of Placental DNA Methylation and Behavioral and Emotional Difficulties in Children at 3 Years of Age

**DOI:** 10.3390/ijms241411772

**Published:** 2023-07-21

**Authors:** Aurélie Nakamura, Lucile Broséus, Jörg Tost, Daniel Vaiman, Silvia Martins, Katherine Keyes, Kim Bonello, Mathilde Fekom, Katrine Strandberg-Larsen, Anne-Laure Sutter-Dallay, Barbara Heude, Maria Melchior, Johanna Lepeule

**Affiliations:** 1 Team of Environmental Epidemiology Applied to Development and Respiratory Health, Institute for Advanced Biosciences (IAB), University Grenoble Alpes, INSERM, 38700 La Tronche, France; lucile.broseus@univ-grenoble-alpes.fr; 2 Laboratory for Epigenetics and Environment, Centre National de Recherche en Génomique Humaine, CEA—Institut de Biologie François Jacob, University Paris Saclay, 91057 Evry, France; tost@cng.fr; 3 From Gametes to Birth, Institut Cochin, U1016 INSERM, UMR 8104 CNRS, Paris Cité University, 75014 Paris, France; daniel.vaiman@inserm.fr; 4 Department of Epidemiology, Columbia University Mailman School of Public Health, New York, NY 10032, USA; ssm2183@cumc.columbia.edu (S.M.); kmk2104@cumc.columbia.edu (K.K.); 5 Institut Pierre Louis d’Epidémiologie et de Santé Publique (IPLESP), Equipe de Recherche en Epidémiologie Sociale (ERES), Sorbonne Université, INSERM, 75571 Paris, France; kim1202@hotmail.fr (K.B.); mathilde.fekom@iplesp.upmc.fr (M.F.); maria.melchior@inserm.fr (M.M.); 6 Department of General Practice, School of Medicine, Sorbonne University, 75013 Paris, France; 7 Section of Epidemiology, Department of Public Health, University of Copenhagen, 1165 Copenhagen, Denmark; ksla@sund.ku.dk; 8 Bordeaux Population Health, Bordeaux University, INSERM, UMR 1219, 33076 Bordeaux, France; alsutter@ch-perrens.fr; 9 University Department of Child and Adolescent Psychiatry, Charles Perrens Hospital, 33000 Bordeaux, France; 10 Center for Research in Epidemiology and Statistics (CRESS), Université Paris Cité and Université Sorbonne Paris Nord, INSERM, INRAE, 75004 Paris, France; barbara.heude@inserm.fr

**Keywords:** EWAS, DNA methylation, DOHAD, placenta, child behavior, pregnancy, epidemiology

## Abstract

The placenta is a key organ for fetal and brain development. Its epigenome can be regarded as a biochemical record of the prenatal environment and a potential mechanism of its association with the future health of the fetus. We investigated associations between placental DNA methylation levels and child behavioral and emotional difficulties, assessed at 3 years of age using the Strengths and Difficulties Questionnaire (SDQ) in 441 mother–child dyads from the EDEN cohort. Hypothesis-driven and exploratory analyses (on differentially methylated probes (EWAS) and regions (DMR)) were adjusted for confounders, technical factors, and cell composition estimates, corrected for multiple comparisons, and stratified by child sex. Hypothesis-driven analyses showed an association of cg26703534 (*AHRR*) with emotional symptoms, and exploratory analyses identified two probes, cg09126090 (intergenic region) and cg10305789 (*PPP1R16B*), as negatively associated with peer relationship problems, as well as 33 DMRs, mostly positively associated with at least one of the SDQ subscales. Among girls, most associations were seen with emotional difficulties, whereas in boys, DMRs were as much associated with emotional than behavioral difficulties. This study provides the first evidence of associations between placental DNA methylation and child behavioral and emotional difficulties. Our results suggest sex-specific associations and might provide new insights into the mechanisms of neurodevelopment.

## 1. Introduction

Behavioral and emotional difficulties of children are expressed as a continuum of symptoms affecting children in their everyday social, school, and family life with various intensities. Behavioral difficulties (also referred to as externalizing symptoms) include, among others, attention deficit hyperactivity disorder (ADHD), conduct problems, and oppositional behavior, while emotional difficulties (or internalizing symptoms) include peer relationship problems, anxiety, and depression [1,2]. About 10 to 15% of preschoolers are estimated to be affected by behavioral or emotional disorders [3,4]. 

In the context of the Developmental Origins of Health and Disease (DOHaD) [5], the first thousand days of life from conception [6] are a critical period for child development, where organs are simultaneously developing and vulnerable to environmental insults, especially during the gestational period [7,8]. Prenatal etiological factors for emotional and behavioral problems in preschoolers include environmental factors such as chemicals and air pollution [9,10,11], alcohol and tobacco consumption [12,13], and psychosocial factors [14]. However, mechanisms underpinning these associations remain poorly understood.

The placenta is the pivotal organ of viviparous species enabling the supply of oxygen and nutrients to the fetus, waste elimination, and fetal hormonal and endocrine regulation [15]. It records a molecular memory of the prenatal environment, in which alterations could be potential biomarkers of fetal exposures [16,17,18,19,20,21] and predictors of health outcomes [22,23]. The placenta is a key organ for fetal and brain development [24,25] and is considered a relevant proxy for brain tissue, with a high number of genes that are expressed at a high level in both placenta and brain tissues [26]. This is well documented with regard to the glucocorticoid and serotonin pathways, which are important for brain maturation and cognitive functions [27]. DNA methylation is the most studied epigenetic mark, especially stable compared to other marks (such as histone code or miRNA profiles), and has been identified as a candidate mechanism underlying child neurodevelopment disorders [20,24]. In some cases, for example, for the tumor suppressor *AHRR*, a decrease in DNA methylation in the promoter region results in an increase in gene expression, while an increase in DNA methylation results in decreased gene expression [28].

A number of placental DNA methylation studies have been conducted with regard to child neurodevelopmental outcomes during the last ten years [22,29,30,31,32,33,34,35]. These studies were mostly conducted on neonates, had relatively small sample sizes, and used hypothesis-driven approaches for the vast majority, targeting a few genes belonging to the hypothalamic–pituitary–adrenal (HPA) axis and to the serotonin pathways [22,29,30,31]. Animal studies showed that dysregulation of glucocorticoids altered fetal brain anatomy and the HPA axis functioning and could predict anxiety in adults [36,37]. Consistently, human placental DNA methylation of the *HSD11B2*, *NR3C1*, and *HTR2A* genes—involved in the HPA and the serotonin pathways—has been shown to be associated with infant neurodevelopment in humans [23]. Males and females present differences in placental DNA methylation levels as well as in the prevalence of behavioral and emotional disorders in childhood [38,39,40], raising the need to explore sex-specific associations.

Epigenome-wide association studies (EWAS) have the potential to uncover other pathways tuning brain wiring, development, and behavior. However, contrary to studies in cord blood [41,42,43,44,45], only one placental EWAS has so far been performed with regard to neonates’ neurobehavior [33]. In this study, conducted on 335 participants, the authors found a statistically significant association between two CpGs within *FHIT* (cg15970800) and *ANKRD11* (cg16710656) and neonate attention. However, to our knowledge, no placental EWAS was conducted on behavioral and emotional difficulties in children, illustrating a gap in the literature. 

We first aimed to explore the associations between placental DNA methylation levels and child behavioral and emotional difficulties using both hypothesis-driven and epigenome-wide approaches on data from 441 mother–child dyads from the EDEN cohort study. Placental DNA methylation was measured using Illumina’s Infinium HumanMethylation450 (450 K) BeadChip, and child emotional and behavioral symptoms were assessed using the four negative subscales of the Strengths and Difficulties Questionnaire (SDQ), involving conduct problems and hyperactivity/inattention (that can be summed up to an externalizing symptoms subscale) and emotional symptoms and peer relationship problems (that can be summed up an internationalizing symptoms subscale). The second aim was to explore potential sex-specific associations by conducting stratified analyses by child sex. 

## 2. Results

### 2.1. Characteristics of Study Participants 

Women were, on average, 29.6 years of age (±4.8 years) at child conception, and about half of them were multiparous. Most women (94%) were Caucasian, and about two-thirds had higher education. Approximately 39% and 22% of women experienced depressive symptoms or adverse events during their pregnancy, respectively. Forty-three percent of women were exposed (either passively or directly) to tobacco smoke during their pregnancy, and 26% were occasional or daily smokers. This sample included 228 boys (52%) and 213 girls (Table 1). 

### 2.2. Emotional and Behavioral Difficulties at 3 years of age

**Figure 1:**
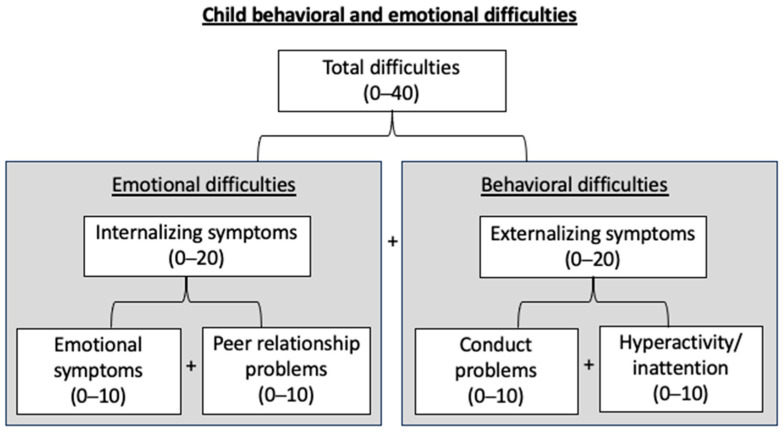
Child emotional and behavioral difficulties assessment at 3 years of age using the four negative subscales of the Strengths and Difficulties Questionnaire (SDQ). Internalizing symptoms = emotional symptoms + peer-relationships problems; externalizing symptoms = inattention/hyperactivity + conduct problems; total = internalizing symptoms + externalizing symptoms.

Emotional and behavioral difficulties in children were assessed by mothers using the four negative subscales (conduct problems, hyperactivity/inattention, emotional symptoms, and peer relationship problems, each subscale being rated out of 10) of the French version of the Strengths and Difficulties Questionnaire (SDQ). Internalizing symptoms (rated out of 20) were defined as the sum of emotional symptoms and peer relationship problems subscales, and externalizing symptoms (rated out of 20) as the sum of conduct problems and hyperactivity/inattention subscales. The addition of internalizing symptoms and externalizing symptoms resulted in a total score (ranging from 0 to 40). In the description and interpretation of the results, “emotional difficulties” can relate to either emotional symptoms, peer relationship problems, or internalizing symptoms, and “behavioral difficulties” to either conduct disorders, inattention/hyperactivity, or externalizing symptoms (Figure 1). 

In this study, internalizing and externalizing symptoms scores, respectively, varied between 0 and 15 and between 0 and 17. Children presented more externalizing (mean score = 6.52/20; sd = 3.60) than internalizing (mean = 3.24/20; sd = 2.41) symptoms (paired Welch test *p*-value < 2.2 × 10^−16^, Supplementary Figure S1A). Boys presented on average more externalizing symptoms and total difficulties than girls (mean externalizing symptoms score = 6.94 for boys vs. 6.06 for girls, Welch test *p*-value = 0.01; mean total difficulties score = 10.11 for boys vs. 9.37 for girls, Welch test *p*-value = 0.11). Their overall level of internalizing symptoms was not significantly different (mean score = 3.17 for boys vs. 3.31 for girls, Welch test *p*-value = 0.57); however, girls presented on average more emotional symptoms (mean score = 1.60 for boys vs. 2.02 for girls, Welch test *p*-value = 0.01) and fewer peer relationship problems than boys (mean score = 1.56 for boys vs. 1.29 for girls, Welch test *p*-value = 0.05) (Table 1). Overall, these results suggest the need to investigate sex-specific associations with regard to DNA methylation levels. The distributions of all SDQ subscales were right-skewed, and 25% of the children presented either no emotional symptoms or peer relationship problems (Supplementary Figure S1B).

The results of all the analyses conducted between placental DNA methylation levels and child emotional and behavioral difficulties at 2 years of age are summarized in Table 2. More precisely, Table 2 reports the number of CpGs associated with each type of symptom and for each type of analysis conducted. Detailed results are provided in the Supplementary Tables mentioned in the following subsections. 

### 2.3. Hypothesis-Driven Analyses 

In the unstratified analyses performed on genes selected from our literature review, one CpG (cg26703534), located on chromosome five, in the body of the *AHRR* gene, was significantly positively associated with the emotional symptoms subscale, after correction for multiple testing (β = 5.85; pFDR = 0.03; average level of methylation = 90%; sd = 4%). The top one hundred CpGs with the smallest *p*-values with regard to child emotional and behavioral symptoms are listed for each of the two hypothesis-driven analyses carried out in Supplementary Tables S1 and S2. The main functions of the genes mentioned in the results are summarized in Supplementary Table S3. In analyses stratified by child sex, no association reached statistical significance after applying an FDR correction for multiple testing. 

### 2.4. Epigenome-Wide Association Study Analyses (EWAS)

**Figure 2:**
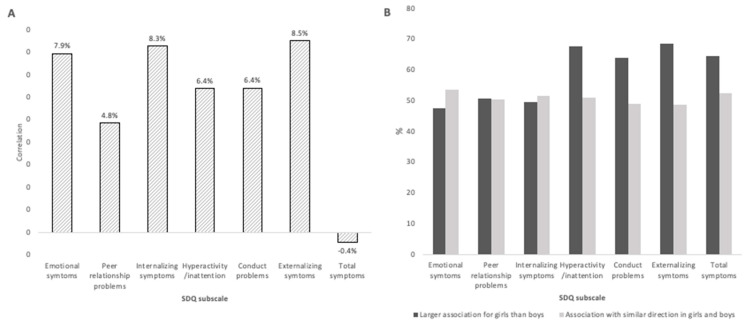
Comparison of EWAS effect sizes in boys and girls using the 371 713 CpGs included in the EWAS. (**A**) Correlation coefficients between beta estimates in girls and in boys; (**B**) Percentage of effects sizes (absolute value betas) that are larger for girls than for boys; percentage of effects sizes with the same direction (either positive or negative) between girls and boys.

As both emotional and behavioral difficulties of the children and placental DNA methylation levels present sex specificities, unstratified EWAS and child sex-stratified EWAS were conducted. In the unstratified EWAS, an increase of one percentage of placental DNA methylation at cg10305789 (0 to 200 bases upstream of the *PPP1R16B* transcription starting site) and cg09126090 (intergenic region) were, respectively, associated with a decrease of 5.85 (pFDR < 0.01; average level of methylation = 28%; sd = 5%) and 5.60 (pFDR = 0.04; average level of methylation = 24%; sd = 6%) in peer relationship problem scores Supplementary Table S4). Among girls, twenty-one CpGs were significantly associated with the emotional symptoms subscale (17 positive and 4 negative associations), and one CpG was significantly positively associated with the internalizing symptoms subscale (Supplementary Table S5). In boys, no association between DNA methylation and child behavior difficulties was statistically significant (Supplementary Table S6). The one hundred CpGs with the smallest *p*-value with regards to any of the SDQ subscales studied differed from girls to boys. Girls’ and boys’ effect sizes were little correlated (between 0 and 8% depending on the SDQ subscale, see Figure 2A). On average, absolute effect sizes related to behavioral and total difficulties were higher in girls than in boys. This tendency was not observed for emotional difficulties effect sizes (Figure 2B).

### 2.5. Differentially Methylated Regional (DMR) Analyses

DMR analysis results are summarized in Table 2 and Table 3 and in Supplementary Table S7. Table 2 describes the number of DMRs significantly associated with each of the SDQ subscales for the DMR analyses unstratified as well as stratified by child sex. The list of the DMRs significantly associated with at least one of the SDQ subscales and the number of CpGs included in the DMR can be found in Table 3 (only DMRs including at least 5 CpGs) and in Supplementary Table S7 (all the DMRs).

Among the 33 DMRs significantly associated with at least one sub-scale of the SDQ, the average number of CpGs per DMR was 8, and 23 DMRs (70%) included at least 5 CpGs (Supplementary Table S7). Among the 33 DMRs, all but 1 (located within *UBXN11*) were positively associated with emotional and behavioral difficulties of the children (see Supplementary Table S8 for the full description of the DMRs). Interestingly, 1 DMR, located within *C6orf47*, was significantly associated with many subscales, including internalizing symptoms (18 CpGs), emotional symptoms (18 CpGs), externalizing symptoms (22 CpGs), hyperactivity/inattention (22 CpGs), and total difficulties (27 CpGs). Among the 23 DMRs containing at least 5 CpGs, 15 were associated with emotional difficulties subscales (either emotional symptoms, peer relationship problems, or internalizing symptoms), including DMRs within *BSCL2*, *CALCB*, *CLIP4*, *EVX1*, and *THSD7A*, which are all protein-coding genes. In addition, 9 DMRs were associated with behavioral difficulties subscales (hyperactivity/inattention, conduct problems, or externalizing symptoms); 2 both with emotional and behavioral difficulties subscales (within *C6orf47* and *ZBBX*) and 1 only with total subscale (Table 3A,B). There were no common DMR between hyperactivity/inattention and conduct problems subscales and between emotional problems and peer relationship problems subscales, suggesting specific associations between DNA methylation and each SDQ subscale (Table 3A,B).

When stratifying our statistical analyses by child sex, we noticed numerous differences that are summarized in Table 3B,C and fully described in Supplementary Tables S9 and S10. In boys, 19 DMRs including at least 5 CpGs (out of 27 DMRs in total—see Supplementary Table S7) were associated with at least one of the SDQ subscales: 7 (4 negatively and 3 positively) with at least one of the emotional difficulties subscales, 7 (2 negatively and 5 positively) with at least one of the behavioral difficulties subscales and 2 (positively) with total problems (Table 3B,C and Supplementary Table S10). In boys, DMRs with the largest number of significantly deregulated CpGs (≥10) associated with emotional difficulties were located within *C5orf13* and *UBXN11*, and the ones associated with behavioral difficulties were located within *GGT1*, *ZNF655*, and within an intergenic region encompassing cg21334513 (Supplementary Table S10). 

Among girls, 49 DMRs with at least 5 CpGs (out of 83 DMRs in total, Supplementary Table S7) were associated with at least one SDQ subscale; including 39 DMRs associated with at least one of the emotional difficulties subscales, 7 with at least one of the behavioral difficulties subscales and 3 with total difficulties subscale. Among the largest DMRs (including at least 10 CpGs), all but two were associated with emotional and total difficulties subscales in boys and all but three were associated with emotional and total difficulties in girls. 

Most of the DMRs were positively associated with emotional and behavioral difficulties in boys as well as in girls. The exceptions were the DMRs associated with peer relationship problems. In boys, all the DMRs with at least 5 CpGs associated with peer relationship problems presented a negative association, and in girls, 5 out of 12 presented a negative association. 

### 2.6. Sensitivity Analyses

In the hypothesis-driven analyses, cg26703534 (*AHRR*) was no longer significantly associated with the emotional symptoms subscale when the association was not adjusted for estimates of cellular heterogeneity, but the beta of the association remained close to the one of the associations with adjustment; furthermore, this was the CpG with the smallest *p*-value with regards to the emotional symptoms subscale (β = 5.36; pFDR = 0.13 without adjustment for cellular composition vs. β = 5.85; pFDR = 0.03 with adjustment for cellular composition, Supplementary Tables S11 and S12). 

In the EWAS, placental DNA methylation at cg10305789 (*PPP1R16B*) remained negatively significantly associated with the peer relationship problems subscale (β = −5.34; pFDR = 0.03). The association between cg09126090 (intergenic region) and peer relationship problems subscale found in the analyses adjusted for cell composition was no longer significant (β = −4.17; pFDR = 0.90), but it belonged to the top 5 hits with the strongest associations with peer relationship problems subscale (Supplementary Table S13). A total number of 31 DMRs were found to be associated with any of the SDQ subscales, and an 81% concordance between the analyses with and without adjustment for cellular composition was estimated (see Supplementary Tables S14 and S15 for the detailed results of the DMR analyses without adjustment for cell mix composition).

## 3. Discussion

Our study, based on the largest to-date sample of placentas collected from the longitudinal EDEN cohort study, identified differentially methylated CpG sites and regions (DMR) with regards to child emotional and behavioral difficulties using both hypothesis-driven and epigenome-wide approaches. Our results mostly show positive associations between placental DNA methylation and child emotional and behavioral symptoms. They suggest that DNA methylation alterations could affect both several domains or could be domain-specific, and could affect either both sexes or could be sex-specific for some genomic regions, with more hits in girls. 

In the hypothesis-driven analyses, children’s emotional symptoms were positively associated with methylation of cg26703534 located in the gene body of the aryl hydrocarbon receptor repressor (*AHRR*) (β = 5.85; pFDR = 0.03). *AHRR* is a known tumor suppressor gene involved in the regulation of cell growth and differentiation, and detoxification pathways. The relationship between the gene body methylation and the expression of the gene remains unclear. Interestingly, in placenta and cord blood, *AHRR* methylation seems to be involved in response to maternal tobacco smoking [28,46,47,48]. This could suggest *AHRR* methylation as a candidate mediator of the association between tobacco smoking exposures during pregnancy and later child emotional and behavioral symptoms [20,46,49]. In adults, decreased blood methylation of *AHRR*, in particular on cg26703534, has been associated with post-traumatic stress disorder cases [50,51]. 

In the EWAS, two CpG sites were significantly negatively associated with peer relationship problems in children: cg09126090, located on an intergenic region on the first chromosome, and cg10305789, located within the promoter region of *PPP1R16B* on the 20th chromosome. *PPP1R16B* is involved in protein phosphatase binding, and its expression has been associated with pre-eclamptic human placentas [52] and maternal hyperglycemia in mice [53]. At the regional level, 33 DMRs were—mostly positively—significantly associated with at least one of the subscales of the SDQ. Twice as many DMRs were associated with emotional difficulties (including emotional symptoms and peer relationship problems) than behavioral difficulties (including inattention-hyperactivity and conduct problems). Only three DMRs were associated with both emotional and behavioral difficulties: within *C6orf47*, an unidentified protein-coding gene located on chromosome six, whose placental methylation has been associated with maternal circadian disruption [54]; *C5orf13*, which may have roles in neural function and cell differentiation [55] and which is highly expressed in brain and placenta; and *UBXN11*, a protein-coding gene involved in the control of cellular processes, such as protein degradation [56]. Previous studies in mouse models suggest that *UBX* family genes are essential for fetal development [57]. In humans, placental *UBXN11* methylation has been associated with lower expression of the gene and with higher birth weight [58]. Within behavioral and emotional difficulties, the fact that there was no overlap between the DMRs associated with inattention/hyperactivity and conduct problems subscales on the one hand and with emotional symptoms and peer relationship problems subscales on the other hand suggest that associations could be domain-specific. DMRs tended to be negatively associated with peer relationship problems. On the opposite, most of the DMRs were positively associated with the other SDQ subscales.

The effect sizes observed in boys were little correlated with the ones observed in girls and tended to be greater in girls than in boys. In the meantime, more DMRs were associated with emotional and behavioral difficulties in girls compared to boys. Only the DMR within *C6orf47* was associated with emotional and behavioral difficulties in both girls and boys. In girls, DMRs were mostly associated with emotional difficulties, whereas in boys, DMRs were as much associated with emotional than behavioral difficulties. Previous studies showed that girls might be more prompt to emotional difficulties, whereas boys were more likely to encounter behavioral difficulties [59]. These results could suggest some sex specificities in terms of DNA methylation marks, both at individual CpG and regional levels, with regards to child emotional and behavioral symptoms at 3 years of age. Sexual dimorphism of the placental methylome [22,38,60,61,62] and of the brain structure and function development [63,64] has been widely documented. Placental dimorphism could be due to distinct effects of sexual hormones and chromosomes [65]. In the current study, we identified for the first time both global and sex-specific differentially methylated probes and regions in the placenta associated with emotional and behavioral difficulties in children. A number of CpGs and DMRs associated with emotional and behavioral difficulties in children did not overlap between boys and girls. Such changes to the placental methylome might partly explain the sex-based differences observed in neurodevelopmental disorders. The placental DNA methylation dimorphism could also result in the distinct transport of environmental pollutants and nutrients through the placenta between male and female fetuses, resulting in a sex-specific response of the fetus [62,66]. Overall, our results provide additional evidence in favor of a mediating role of the placental methylome in the developmental origins of health and diseases [67,68].

Interestingly, in our unstratified analyses, we identified some DMRs that have been previously involved in mental health issues. The emotional symptoms subscale was significantly positively associated with DMRs located within *EVX1*, which is involved in central nervous system development, and *THSD7A*, which has been shown to be highly expressed in the placenta and could play an important role in the appropriate vascularization of the placenta [69]. Placental DNA methylation of *CALCB*, involved in hormone and neuropeptide hormone activity, and *CLIP4*, a biomarker of suicidal ideation [70], were also associated with the emotional symptoms subscale in our unstratified analyses as well as in girls for *CLIP4*. The peer relationship problems subscale was significantly positively associated with DMRs located near *BSCL2* (in unstratified analyses and in girls), involved in the adipogenesis and glucose/energy metabolism pathways. The hyperactivity/inattention subscale was significantly associated with DMRs located near the *FAM3B* gene (in unstratified analyses and in boys), which is involved in cytokine activity, and *ProSAPiP1*, *RUFY2* (both in unstratified analyses and in boys) and *ZBBX* (only in unstratified analyses) genes, previously identified as a candidate susceptibility genes for ADHD [71]. Regarding prenatal exposures, *ZBBX* placental methylation was found to be positively associated with maternal triclosan exposure in boys from the EDEN cohort [19]. Furthermore, DMRs within *GGT1* and *ZNF655*, associated with behavioral difficulties, were previously found to be associated with maternal tobacco smoking during pregnancy [72] and smoking status in adults [73]. 

In a previous EWAS conducted on a sample of 335 mother–neonate dyads, Paquette and colleagues [33] identified a statistically significant positive relationship between placental DNA methylation at a CpG located within *FHIT* and newborn attention and a negative relationship between placental DNA methylation of *ANKRD11* and neonate attention scores, measured using the NNNS scale and without correction for multiple testing. Interestingly, these CpG sites within the *FHIT* and *ANKRD11* genes belonged to the top 100 CpGs associated with child emotional and behavioral development in our hypothesis-driven approach, but they were not statistically significant after correction for multiple testing. The SDQ and the NNNS evaluate different domains of child development at different ages: 3 years of age for the SDQ in our study versus newborn in Paquette and colleagues, which can explain the small overlap between the two studies. 

Our study is the so far only study that characterized the associations between placental DNA methylation and child behavior among preschoolers. It presents several strengths. Standardized tools were used for assessing child behavior and adjusting our analyses for the major confounding factors in the association between DNA methylation and child behavior. Whether adjusting EWAS for cell heterogeneity is relevant is still debated [74,75]. Epigenetic marks are highly specific to cell types in the placenta as in other tissues. Therefore, part of the DNA methylation levels measured are driven by the cell composition of the tissue sample [76]. Our analyses were adjusted for cell heterogeneity [28], using placenta reference-based estimates [77] that showed better performance than reference-free estimates [78]. Adjusting the analyses on cellular composition estimates had little effect on our conclusions. Finally, conducted analyses were stratified by child sex in order to take into account potential sex-specificities in the association of DNA methylation with child behavior [38,79]. 

Child emotional and behavioral problems were not assessed by a clinical evaluation but by the Strengths and Difficulties Questionnaires [80]. However, this scale has shown acceptable performances for assessing emotional and behavioral problems in school-aged children. The SDQ has notably been validated among preschoolers in its Swedish version [81]. We might lack statistical power to detect significant associations between placental DNA methylation at a CpG level and child emotional and behavioral difficulties. In order to overcome the lack of statistical power, differentially methylated regions analyses were conducted in addition to CpG-specific analyses. Most of the DMRs included a high number of CpGs (on average, eight CpG per DMR in the unstratified analyses), which reinforces the robustness of our results. One perspective of this study would be to include larger sample size, by conducting a meta-analysis using children from birth cohorts consortia, for example [20]. 

## 4. Materials and Methods

### 4.1. Study Population

**Figure 3:**
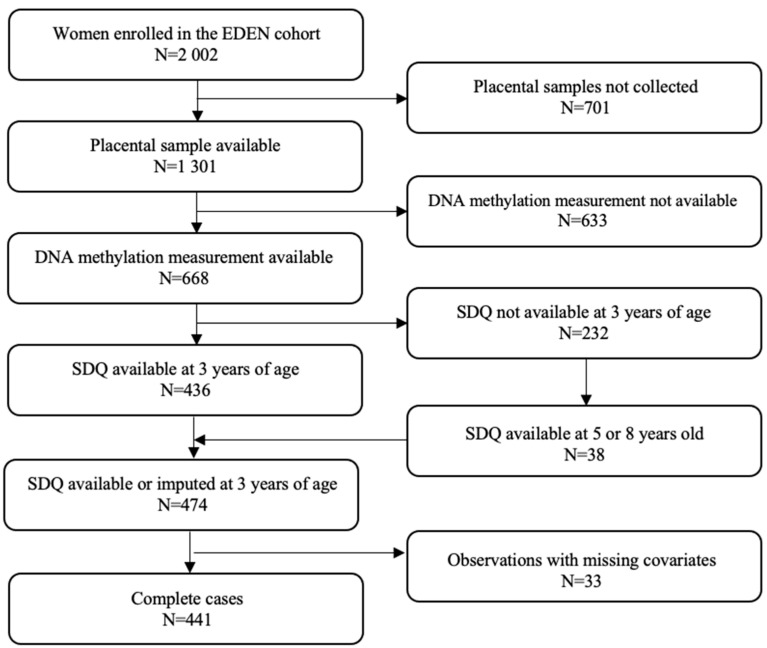
Flow chart of the study; SDQ: Strengths and Difficulties Questionnaire.

Mothers and children included in this study are a subset of the EDEN Mother–Child Cohort study participants [82]. Between 2003 and 2006, 2002 pregnant women were recruited before 24 weeks of gestation in the university hospitals of Nancy and Poitiers, France. Exclusion criteria included multiple pregnancies, pre-pregnancy diabetes, French illiteracy, and plans to move outside the region within the following 3 years. Lifestyle, demographic and medical data were collected by questionnaires either completed directly by participating women or administered by midwives during clinical examinations between 24 and 28 weeks of gestation, and additionally at 4 months, 8 months, 1 year, 2 years, 3 years, 5.5 years, and at 8 years post-delivery. DNA methylation (DNAm) was measured in placental samples from 668 women included in the EDEN cohort. Among women with measures of DNAm, children’s behavioral and emotional difficulties were available for 474 participants. Finally, our study population included 441 dyads without missing values on any of the covariates (Figure 3). The EDEN cohort study received approval from the ethics committee (CCPPRB) of Kremlin Bicêtre and from the French data privacy institution Commission Nationale de l’Informatique et des Libertés (CNIL). Written consent was obtained from the mother for herself and for the offspring.

### 4.2. Child’s Emotional and Behavioral Difficulties at 3 Years of Age

The child’s behavioral and emotional difficulties were assessed by mothers using the French version of the Strengths and Difficulties Questionnaire (SDQ). Translated into several languages, the SDQ is a validated screening tool for emotional and behavioral problems in children and adolescents from 3 years of age [80,83]. The SDQ includes 25 items divided into 5 subscales of 5 items each: one positive subscale covering pro-social behavior (not included in our analyses) and four negative subscales involving conduct problems, hyperactivity/inattention, emotional symptoms, and peer relationship problems. Each subscale is scored from 0 to 10. The addition of the 4 negative subscales generates a total difficulties score ranging from 0 to 40. In accordance with the general instructions of the use of the SDQ [84], internalizing symptoms were defined as the sum of emotional symptoms and peer relationship problems subscales (score varying from 0 to 20) and externalizing symptoms as the sum of conduct problems and hyperactivity/inattention subscales (score varying from 0 to 20) [83]. All the subscales we used in our analyses are summarized in Figure 1. As trends in child behavior appear to be stable over time [1], including in EDEN [85], missing values for child SDQ at 3 years of age were imputed, when available, by the one measured at 5 or 8 years of age (N = 38, Figure 3).

### 4.3. Placental DNA Methylation Levels

Placental biopsies were collected at the fetal side of each placenta at delivery by the midwife or the technician of the study using a standardized procedure. DNA extraction and methylation assessment were previously described elsewhere [16]. Briefly, DNA concentration was determined by Nanodrop measurement and fluorescent quantification using Picogreen by the Centre National de Recherche en Génomique Humaine (CNRGH, Evry, France). DNA samples were plated onto nine plates, including 64 chips, that were analyzed in 4 batches. Each chip was balanced according to child sex and recruitment center. The Illumina’s Infinium HumanMethylation450 (450 K) BeadChip, which represents over 485,000 individual CpG sites, was used to assess the levels of methylation in placenta samples, following the manufacturer’s instructions (Illumina, San Diego, CA, USA). Raw signals were extracted using the GenomeStudio^®^ software (version 2011.1. Illumina). For each CpG, DNA methylation level was calculated as the ratio of the intensity of fluorescent signals of the methylated alleles over the sum of methylated and unmethylated alleles (β value, ranging from 0 (unmethylated) to 1 (fully methylated)). All samples passed initial quality control and had, on average, more than 98% of valid data points (detection *p*-value < 0.01). DNA methylation data were corrected and normalized using a Beta MIxture Quantile (BMIQ) normalization method, which corrects for probe type differences [86]. 

### 4.4. Covariates

Covariates were a priori identified based on a literature review and a Directed Acyclic Graph (DAG) and included: the mother’s age at conception (as a continuous variable), mother’s ethnicity (Caucasian; Other—estimated using the planet R package version 1.8.0 [87]), parity (0; ≥1 other child), maternal educational attainment (≤20; >20 years old), adverse events during pregnancy (none; at least one among fourteen adverse events including separation or divorce; death or serious illness of a close friend or a family member; being evicted from accommodation; deal with a fire, a flood or a major disaster in the dwelling; being involved in a serious traffic accident; losing job; legal issues; having a partner facing: job loss; legal issues; alcohol-related problem; as well as being hit or brutalized; forced to have sexual intercourses or being harassed by the partner or finally have faced another disturbing event). Covariates also included depressive symptoms during pregnancy, assessed via the Centre for Epidemiological Studies Depression (CES-D) questionnaire at 24 weeks of amenorrhea [88], using a cut-off of 16 and above [89], maternal tobacco exposure during pregnancy (no exposure; passive smoking; mother smoking), child sex (boy; girl), technical factors related to DNAm measurements: batch, plate, and chip (Supplementary Figure S2).

### 4.5. Cellular Heterogeneity of Placenta Samples 

DNA methylation levels differ among cell types within the placenta [90]. Some proportion of the DNA methylation variability observed across samples may be due to the interindividual heterogeneity of the placenta samples. Reference-based [91] placental cellular composition was estimated using DNA methylation data based on Yuan’s method, implemented in the planet R package version 1.8.0 [77,87]. Briefly, using their results as reference methylation profiles of the six major human term placental cell types (Endothelial, Hofbauer, nRBC, Stromal, Syncytiotrophoblast, and Trophoblasts), the cellular composition was estimated in our data using a Robust Partial Correlations (RPC) cell-type deconvolution algorithm implemented in the R package EpiDISH version 2.16.0. 

### 4.6. Statistical Analyses

**Figure 4:**
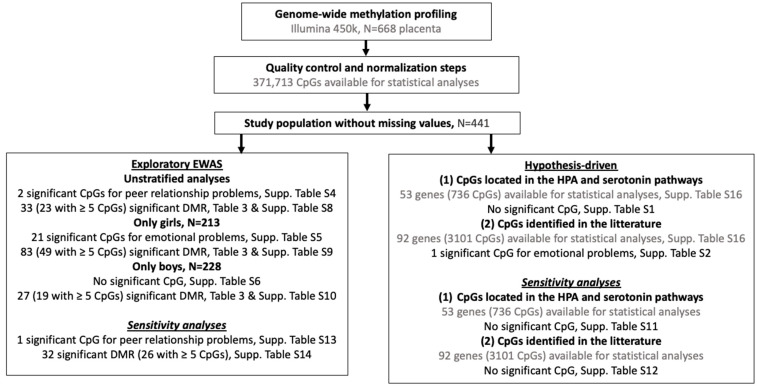
Workflow of the study; EWAS = epigenome-wide association study; DMR = differentially methylated region; Significance at pFDR < 0.05 for the hypothesis-driven and EWAS and pSidak < 0.05 for the DMR analyses.

A workflow of all the statistical analyses is provided in Figure 4. Conduct problems, hyperactivity/inattention, externalizing symptoms, and total difficulties SDQ subscales were modeled using quasi-Poisson regressions due to their right-skewed distributions and overdispersion [92] whereas emotional symptoms, peer relationship problems, and internalizing subscales were modeled using a negative binomial regression because of the excess of zeros. Multivariate regressions were adjusted for the covariates listed above in addition to cellular composition and stratified by child sex. As few mother–child dyads had few missing values on covariates (7%), we decided not to impute missing data. 

Hypothesis-driven analyses were based on: (i) 53 genes (corresponding to 736 CpGs) identified to be involved in the hypothalamic-pituitary-adrenal axis (HPA) [93] and the dopamine and serotonin [94] dysregulation in relation to neurodevelopmental disorders (ii) 92 genes (3 101 CpGs) associated with child neurodevelopment in previous hypothesis-driven and epigenome-wide studies (Supplementary Table S16). The *p*-values were given with a correction for multiple testing using the Benjamini and Hochberg false discovery rate (FDR) procedure [95]. An FDR value < 0.05 was considered statistically significant.

In parallel, we carried out an exploratory epigenome-wide association study (EWAS) unstratified and stratified by child sex. Analyses were performed on a total of 371,713 CpGs that passed quality control, with the aim of discovering new genes/genomic regions where placental DNA methylation is associated with child behavioral and emotional difficulties. CpGs located on X and Y chromosomes were excluded. For the EWAS approach, *p*-values were corrected for multiple testing using the FDR procedure. An FDR value < 0.05 was considered statistically significant. Differentially methylated regions (DMR) were identified using the comb-p method [96]. These regional analyses allow a gain in statistical power to detect changes in DNAm versus single CpG site analyses [97]. The *p*-values obtained by the EWAS analyses were combined using sliding windows that account for spatial correlations across the genome using Stouffer-Liptak-Kechris correction [98]. DMR’s *p*-values were adjusted for multiple testing using the Šidác correction [99], with a threshold of 0.05. Significant DMRs included at least two probes (*p*-value < 0.001 to start a region) at a maximum distance of 500 bp. Probes and DMRs were characterized using the GeneCards Human Gene Database [100].

### 4.7. Sensitivity Analyses

Whether interindividual variation in methylation levels is dependent or independent of cellular heterogeneity is impossible to distinguish [74]. Therefore, we conducted a sensitivity analysis replicating both hypothesis-driven and epigenome wide analyses on each SDQ subscale without adjustment for cellular composition.

### 4.8. Softwares

All the statistical analyses were conducted using R Studio 1.3.1093 version (PBC, Boston, MA, USA). Quasi-Poisson and negative binomial regressions were computed using the glm and glm.nb functions from the Stats and MASS (version 7.3-60) R packages, respectively. DMR analyses were computed using the comb-p method [96], implemented in the ENmix R package version 1.36.01. 

## 5. Conclusions

In this study, we showed that among girls, placental DNA methylation was mostly associated with internalizing symptoms, both at an individual CpG and at a regional level, whereas in boys, DMRs were as much associated with internalizing than externalizing symptoms. Our results further suggest sex-specific associations between placental DNA methylation and child emotional and behavioral difficulties. Further analyses would be necessary to confirm our results. 

## Figures and Tables

**Table 1:** Description of the study population. Italics in all the columns + “*Mean*” and “*sd*” labels for quantitative variables have been used to distinguish *means* from N and *sd* from %.

N = 441	N or *Mean*	% or *sd*
**Emotional and behavioural difficulties**		
Emotional symptoms	*1.8*	*1.7*
Girls	*2.0*	*1.8*
Boys	*1.6*	*1.7*
Peer relationship problems	*1.4*	*1.5*
Girls	*1.3*	*1.3*
Boys	*1.6*	*1.6*
Internalizing symptoms	*3.2*	*2.6*
Girls	*3.3*	*2.4*
Boys	*3.2*	*2.8*
Conduct problems	*3.2*	*2.0*
Girls	*2.9*	*1.9*
Boys	*3.4*	*2.0*
Inattention/hyperactivity	*3.4*	*2.2*
Girls	*3.1*	*2.1*
Boys	*3.6*	*2.3*
Externalizing symptoms	*6.5*	*3.6*
Girls	*6.1*	*3.4*
Boys	*6.9*	*3.7*
Total symptoms	*9.8*	*4.9*
Girls	*9.4*	*4.6*
Boys	*10.1*	*5.1*
**Sociodemographic characteristics**		
Mother’s age at conception (years)	*29.6*	*4.8*
Parity		
No other child	206	0.47
At least one other child	235	0.53
Mother’s ethnicity		
Caucasian	415	0.94
Other	26	0.06
Mother’s educational attainment		
Low	139	0.32
High	302	0.68
**Pregnancy characteristics**		
Depressive symptoms during pregnancy		
No (CES-D < 16)	343	0.78
Yes (CES-D >=16)	98	0.22
Adverse events during pregnancy		
None	270	0.61
At least one	171	0.39
Maternal tobacco smoking exposure during pregnancy		
Non-smoker or former smoker and no secondhand	251	0.57
Non-smoker or former smoker and secondhand	74	0.17
Occasional or smoker during all pregnancy	116	0.26
**Infant characteristics**		
Sex		
Boy	228	0.52
Girl	213	0.48
**Estimated cellular composition**		
Endothelial	*0.10*	*0.03*
Hofbauer	*0.02*	*0.01*
nRBC	*0.02*	*0.01*
Stromal	*0.11*	*0.03*
Syncytiotrophoblast	*0.64*	*0.08*
Trophoblasts	*0.13*	*0.06*

**Table 2:** Summary of the hypothesis-driven, EWAS, and DMR analyses—Number of significant associations between placental DNA methylation and child behavior; pFDR < 0.05 for the hypothesis-driven and epigenome-wide association studies and pSidak < 0.05 for the differentially methylated regions analyses; + positive association; − negative association; CC = cellular composition, EWAS = epigenome-wide association study, DMR = differentially methylated region. Internalizing symptoms subscale = emotional symptoms subscale + peer-relationships problems subscale; externalizing symptoms subscale = inattention/hyperactivity subscale + conduct problems subscale; total = internalizing symptoms subscale + externalizing symptoms subscale (indicated by different colors).

	Emotional Difficulties			Behavioural Difficulties			
Approach/Subscale	Emotional Symptoms	Peer Relationship Problems	Internalizing symptoms	Inattention/ Hyperactivity	Conduct Problems	Externalizing Symptoms	Total
Hypothesis driven							
HPA/Serotonin pathways (53 genes—736 CpGs)	0	0	0	0	0	0	0
Literature review (92 genes—3 101 CpGs)	1+	0	0	0	0	0	0
EWAS	0	2−	0	0	0	0	0
Girls	17+/4−	0	1+	0	0	0	0
Boys	0	0	0	0	0	0	0
DMR	11	5	12	5	4	4	12
Girls	48	12	17	7	3	0	11
Boys	4	4	9	5	4	0	9
EWAS without CC adjustment	0	1−	0	0	0	0	0
DMR without CC adjustment	13	4	7	6	5	5	12

**Table 3:** Number of CpGs within the identified differentially methylated regions (DMR) with at least five CpGs associated with child SDQ at 3 years of age—Unstratified and stratified analyses on child sex. * DMR significantly associated with at least one of the emotional difficulties subscales (emotional symptoms, peer relationship problems, and internalizing symptoms); † DMR significantly associated with at least one of the behavioral difficulties subscales (hyperactivity/inattention, conduct problems, and externalizing symptoms); NA = intergenic region. Internalizing symptoms subscale = emotional symptoms subscale + peer-relationships problems subscale; externalizing symptoms subscale = inattention/hyperactivity subscale + conduct problems subscale; total = internalizing symptoms subscale + externalizing symptoms subscale (indicated by different colors).

	All Children (N = 441)							Only Boys (N = 228)							Only Girls (N = 213)						
DMR	Emotional Symptoms	Peer relationship Problems	Internalizing Symptoms	Hyperactivity/Inattention	Conduct Problems	Externalizing Symptoms	Total Symptoms	Emotional Symptoms	Peer relationship Problems	Internalizing Symptoms	Hyperactivity/Inattention	Conduct Problems	Externalizing Symptoms	Total Symptoms	Emotional Symptoms	Peer relationship Problems	Internalizing Symptoms	Hyperactivity/Inattention	Conduct Problems	Externalizing Symptoms	Total Symptoms
**(A) DMRs only found in unstratified analyses (N = 9)**																					
*ANKS1B* *		6																			
*BIN2* *			7																		
*CALCB* *	6																				
*EVX1* *	6		6				6														
*NPY* *			6																		
*ProSAPiP1* †				9																	
*THSD7A* *	7		7				8														
*VWCE* *			8				11														
*ZBBX* *†		11		9																	
**(B) DMRs found both in unstratified and stratified analyses by child sex (N = 14)**																					
*BSCL2* *		9														9					
*C3orf26* *	6		6					5													
*C5orf13* *	13		12				13	13		7											
*C6orf47* *†	18		18	22		22	27							18				28			25
*CCDC62*							9											8			8
*CLIP4* *	8														8						9
*GGT1* *			9				9					10		9							
*GPR75* †			9												9						
*FAM3B* †				8		8	7				7										
*MIR564* †					12														13		
*RUFY2* †				8														7			
*THBD* †					6							7									
UBXN11*		5							12	11											
*ZNF844* †					6	6	6												7		
**(C) Sex-specific DMRs (N = 54)** (** No significant DMR)																					
ACP 5*										5											
*ADAMTS17* *															11		6				
*B4GALNT2* *															7		5				
*C6orf52* *																5					
*C10orf25* *																	10				
*C16orf67* *																5					
*C17orf46* *									7	6											
*CCNA1* *															5						
*CROT* *															12						
*CWH43* *															8						
*EN1*															5						
*FLJ42289* *															6						
*FLOT1* *																	11				8
*GNAS* *															10						
*HECW1* *																6					
*HFE* *															6						
*HLA-F* *															10						
HORMAD 2*																9					
*IGFBP3* *															8		7				
*KIAA0101*														8							
*LOC148824* *																5					
*LOC641518* *															5						
*MECOM* *																5					
*MOCS1*														8							
*NA (*cg04373548*)* †																			8		
*NA (*cg05931423*)* *																5					
*NA (*cg18081456*)* *																	5				
*NA (*cg18356974*)*														5							
*NA (*cg21334513*)* †												12									
*NA (*cg27405554*)*																					5
*NR2E1* *																9					
*PAX6* *																	10				
*PRRT1* *																14					
*RIN2*																					5
*RPS6KA2* †											9										
*SEC14L4* *										7											
*SERPING1* *																6					
*SOX11* *																5	5				5
*SP9* †											6										
*STAM* †											6			6							
*TBX15* *																	6				
*TEKT1*																					6
*TM6SF1* *																5					
*TMEM176B* *																6					
*TRIM26* *																10					
*TRIM72* *																14					
*TSPYL3* *																13					
*TSTD1* *																	10				
*WSCD2* *								5		5				5							
*ZFP42* *																9					
*ZMYND10* †																		11			
*ZNF655*														10							
*ZNF780B* *																5					
*ZSCAN16* *																	8				

## Data Availability

The data presented in this study are available on request from etude.eden@inserm.fr.

## Supplementary Materials

The supporting information can be downloaded at: https://www.mdpi.com/article/10.3390/ijms241411772/s1.
