# Supplementary material for: Epigenome-Wide Associations of Placental DNA Methylation and Behavioral and Emotional Difficulties in Children at 3 Years of Age
Source: Int J Mol Sci. 2023 Jul 21;24(14):11772. doi: 10.3390/ijms241411772 (PMC10380531; doi:10.3390/ijms241411772)
Supplement: Supplementary file 1 [file ijms-24-11772-s001.zip › Supplementary Figures S1-S2.pdf]

A. All children together (N=441)

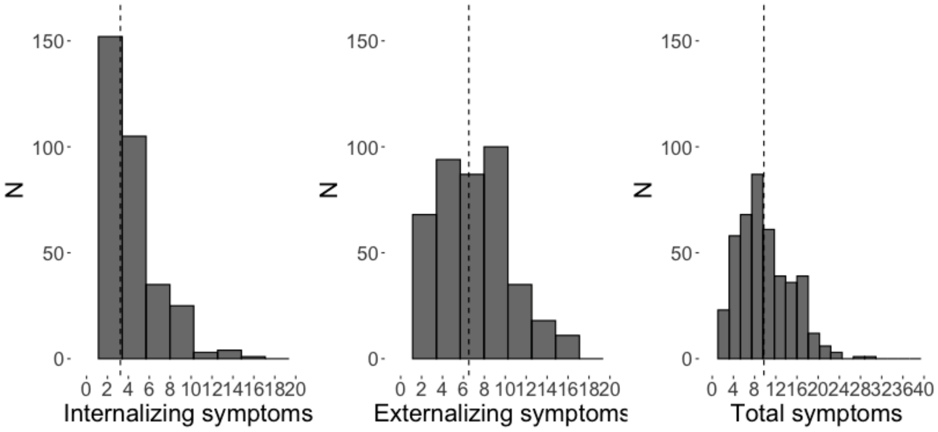

B. Cohort stratified by child sex (N=228 boys and N=213 girls)

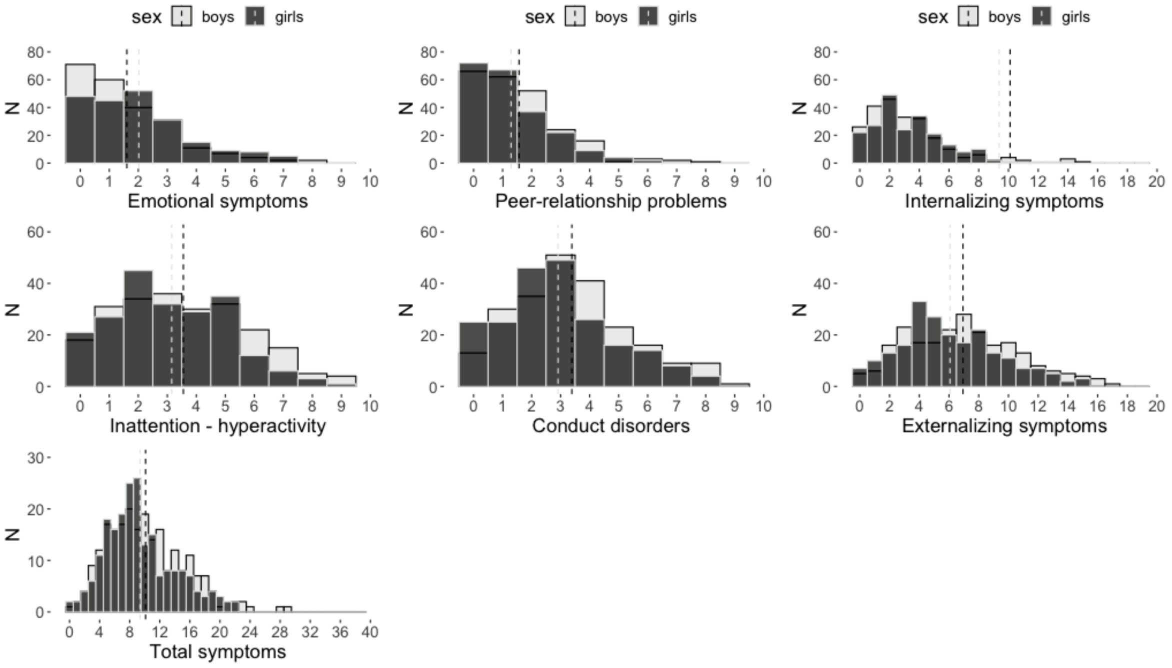

Supplementary Figure S1. Strengths and Difficulties Questionnaire (SDQ)'s subscales distributions (n = 441); Dash lines correspond to average values of each SDQ subscale for girls and boys.

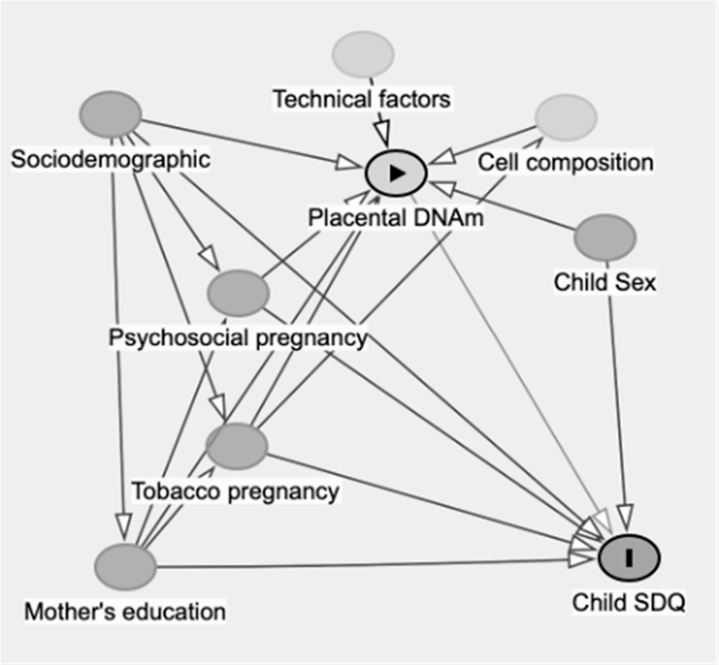

Supplementary Figure S2. Directed Acyclic Graph (DAG) for the selection of the covariates in the multivariate analyses between placental DNA methylation and child SDQ at 3 years of age; DNam = DNA methylation; SDQ = Strengths and Difficulties Questionnaire (at 3 years of age); Sociodemographic factors include mother's age at conception, parity and mother's ethnicity; psychosocial factors during pregnancy include depressive symptoms and adverse events during pregnancy and technical factors include batch, plate and chip.
